# Decreased Levels of Foldase and Chaperone Proteins Are Associated with an Early-Onset Amyotrophic Lateral Sclerosis

**DOI:** 10.3389/fnmol.2017.00099

**Published:** 2017-04-06

**Authors:** Melania Filareti, Silvia Luotti, Laura Pasetto, Mauro Pignataro, Katia Paolella, Paolo Messina, Elisabetta Pupillo, Massimiliano Filosto, Christian Lunetta, Jessica Mandrioli, Giuseppe Fuda, Andrea Calvo, Adriano Chiò, Massimo Corbo, Caterina Bendotti, Ettore Beghi, Valentina Bonetto

**Affiliations:** ^1^Istituto Di Ricerche Farmacologiche Mario Negri, Istituti di Ricovero e Cura a Carattere Scientifico (IRCCS)Milan, Italy; ^2^Department of Neurorehabilitation Sciences, Casa Cura PoliclinicoMilan, Italy; ^3^Center for Neuromuscular Diseases and Neuropathies, Unit of Neurology, ASST Spedali Civili and University of BresciaBrescia, Italy; ^4^NEuroMuscular Omnicentre, Fondazione Serena OnlusMilan, Italy; ^5^Department of Neuroscience, Azienda Ospedaliero Universitaria di Modena, Ospedale Civile S. Agostino-EstenseModena, Italy; ^6^ALS Center, Department of Neuroscience Rita Levi Montalcini, University of TorinoTorino, Italy

**Keywords:** protein folding, response to stress, chaperone, biomarkers, foldase

## Abstract

Amyotrophic lateral sclerosis (ALS) is a fatal neurodegenerative disease characterized by a progressive upper and lower motor neuron degeneration. One of the peculiar clinical characteristics of ALS is the wide distribution in age of onset, which is probably caused by different combinations of intrinsic and exogenous factors. We investigated whether these modifying factors are converging into common pathogenic pathways leading either to an early or a late disease onset. This would imply the identification of phenotypic biomarkers, that can distinguish the two populations of ALS patients, and of relevant pathways to consider in a therapeutic intervention. Toward this aim a differential proteomic analysis was performed in peripheral blood mononuclear cells (PBMC) from a group of 16 ALS patients with an age of onset ≤55 years and a group of 16 ALS patients with an age of onset ≥75 years, and matched healthy controls. We identified 43 differentially expressed proteins in the two groups of patients. Gene ontology analysis revealed that there was a significant enrichment in annotations associated with protein folding and response to stress. We next validated a selected number of proteins belonging to this functional group in 85 patients and 83 age- and sex-matched healthy controls using immunoassays. The results of the validation study confirmed that there was a decreased level of peptidyl-prolyl *cis-trans* isomerase A (also known as cyclophilin A), heat shock protein HSP 90-alpha, 78 kDa glucose-regulated protein (also known as BiP) and protein deglycase DJ-1 in PBMC of ALS patients with an early onset. Similar results were obtained in PBMC and spinal cord from two SOD1^G93A^ mouse models with an early and late disease onset. This study suggests that a different ability to upregulate proteins involved in proteostasis, such as foldase and chaperone proteins, may be at the basis of a different susceptibility to ALS, putting forward the development of therapeutic approaches aiming at boosting the protein quality control system.

## Introduction

Amyotrophic lateral sclerosis (ALS) is a fatal neurodegenerative disease characterized by a progressive upper and lower motor neuron degeneration. ALS is a highly heterogeneous disorder, with phenotypic variability involving a number of clinical aspects such as site of onset, age of onset, rate of progression, nonmotor involvement and response to therapy (Beghi et al., 2011). This makes it difficult to decipher pathogenesis and to develop therapeutic strategies, also because biomarkers that reflect heterogeneity and may be useful for stratification are missing. A combination of genetic and environmental/exogenous modifying factors are believed to underlie this variability. The identification of the pathways affected by the modifying factors is of great interest, as they may be targets of therapeutic intervention.

Age is a known risk factor for ALS. The majority of ALS patients have disease onset between 55 and 75 years of age, however patients with an age of onset <55 years and >75 years do exist and represent, respectively, the 22% and the 16% of all patients, as recently reported in a large multicenter Italian study (Calvo et al., 2016). Several factors, both genetic and environmental/exogenous, have been reported to modify age of onset in patients and animal models of ALS. FUS mutations have been frequently associated with early-onset disease in familial and sporadic cases (Chiò et al., 2009; Hübers et al., 2015). Earlier onset of ALS has been also observed in soldiers and professional soccer players (Haley, 2003; Chiò et al., 2005; Pupillo et al., 2012). In these patients, an anticipated onset might be linked to heavy physical exercise, repeated trauma, and/or exposure to as yet unknown toxic agents in genetically predisposed individuals (Beghi and Morrison, 2005; Pupillo et al., 2012; Beghi, 2013). A mutant SOD1^G93A^ mouse model of ALS on a 129Sv genetic background has an earlier age of onset than a SOD1^G93A^ mouse model on a C57BL6 genetic background despite an equal expression of the mutant protein (Marino et al., 2015). These mice have also faster disease progression and an intrinsic marked down-regulation of specific pathways involved in mitochondrial function and protein quality control (Nardo et al., 2013, 2016; Marino et al., 2015). Conversely, ApoE2 polymorphism and lower EPHA4 expression have been correlated with delayed age of onset in ALS patients (Li et al., 2004; Van Hoecke et al., 2012). Moreover, in mutant SOD1 mice female showed a later onset than male and this delay further increased with exercise (Veldink et al., 2003).

Summarizing, different combinations of intrinsic and exogenous factors, that can be hardly discerned, may lead to an anticipated or a delayed onset of the disease. We wanted to test whether or not modifying factors are converging into common pathogenic pathways leading either to an early or a late disease onset. This would imply the identification of phenotypic biomarkers, that can distinguish the two populations of ALS patients, and of relevant pathways to consider in a therapeutic intervention. Toward this aim a significant cohort of incident ALS patients with either early (<55 years of age) or late (>75 years of age) disease onset was recruited from population-based Italian registries, and several analyses were performed. We found that an epigenetic rearrangement such as whole-blood DNA methylation could not distinguish the two groups of ALS patients (Tremolizzo et al., 2014). However, difference in plasma amino acid levels were observed in relation to the age of onset (Cecchi et al., 2014). In this work, we identified protein phenotypic biomarkers in peripheral blood mononuclear cells (PBMC) of the same cohort of patients indicating that protein levels in PBMC could be good biological correlates of the age of onset in ALS. The same phenotypic biomarkers were also analyzed in the two mutant SOD1 mouse models of ALS with early and late disease onset indicating common changes between the sporadic and SOD1-linked forms. These biomarkers are proteins involved in the maintenance of protein homeostasis, or proteostasis. Proteostasis is attained through several quality-control systems that assist protein folding, clear misfolded proteins and respond to protein aggregation, whose major players are molecular chaperones. Interestingly, impaired proteostasis has emerged as a key contributor to the pathogenesis of ALS (Ruegsegger and Saxena, 2016).

## Materials and Methods

### Antibodies

The antibodies for Western and dot blot, used for human and mouse samples, were: rabbit polyclonal anti-peptidyl-prolyl *cis-trans* isomerase A (PPIA) antibody (1:2500 dilution; Millipore), rabbit polyclonal anti-heat shock protein HSP 90-alpha (HSP90) (1:3000 dilution; Stressgen), rabbit polyclonal anti-78 kDa glucose-regulated protein (GRP78) (1:500 dilution; Santa Cruz Biotechnology Inc.), mouse monoclonal anti-endoplasmic reticulum protein 57 (ERp57) (1:2500 dilution; Stressgen), rabbit monoclonal anti-protein deglycase DJ-1 (DJ-1) (1:1500 dilution; Abcam), mouse monoclonal anti-heat shock cognate 71 kDa protein (HSC70) (1:1000 dilution; Santa Cruz Biotechnology Inc.), rabbit polyclonal anti-TDP-43 (1:2500 dilution; Proteintech), goat anti-mouse or anti-rabbit peroxidase-conjugated secondary antibodies (1:5000; Santa Cruz Biotechnology Inc.).

### Subjects

Following approval of the protocol by the ethics committees (Comitato Etico Interaziendale A.O.U. San Giovanni Battista di Torino – A.O. “C.T.O. Maria Adelaide di Torino”, Torino, Italy; Comitato etico degli Spedali Civili di Brescia, Brescia, Italy; Comitato Etico Provinciale di Modena, Modena, Italy) written informed consent was obtained from all participating subjects. Patients, enrolled in three Italian population-based registries (Lombardia, Piemonte, Emilia Romagna), were newly diagnosed definite, probable or possible ALS, according to the El Escorial criteria (Brooks, 1994). To be eligible, patients had to be ≤55 years of age (early ALS) or ≥75 years of age (late ALS) at diagnosis. Controls were residency-, sex-, and age-matched (±5 years), randomly chosen within the same hospital of the patient among subjects admitted for surgery for a non-spontaneously evolving disease. Subjects enrolled for the discovery phase study were 16 early ALS, 16 late ALS and 32 controls (early and late controls), with demographic and clinical characteristics described in **Table 1**. Subjects enrolled in the validation study were 85 ALS patients and 83 controls with demographic and clinical characteristics described in **Table 2**. All cases were sporadic. A wide, although partial screening for ALS-causative mutations was performed (Tremolizzo et al., 2014). Two early ALS cases carried a TARDP mutation (M359V, G368S), one late ALS case carried a SOD1 mutation (D90A) and another one an OPTN mutation (L500P).

**Table 1:** Demographic and clinical characteristics of the enrolled population in the proteomic study.

Residency	Diagnosis	Age^a^	Sex	Form^b^	Score^c^	Duration^d^	*N*
Lombardia	Early ALS	50 ± 5	4(M), 4(F)	8(S)	30 ± 11	16 ± 7	8
	Early Controls	48 ± 5	4(M), 4(F)	n/a	n/a	n/a	8
	Late ALS	78 ± 2	4(M), 4(F)	4(S), 1(B), 3(–)	29 ± 10	14 ± 5	8
	Late Controls	77 ± 4	2(M), 6(F)	n/a	n/a	n/a	8
Piemonte	Early ALS	47 ± 6	2(M), 2(F)	1(S), 3(B)	37 ± 11	15 ± 4	4
	Early Controls	49 ± 7	2(M), 2(F)	n/a	n/a	n/a	4
	Late ALS	81 ± 4	3(M), 1(F)	3(S), 1(B)	33 ± 9	15 ± 4	4
	Late Controls	79 ± 4	3(M), 1(F)	n/a	n/a	n/a	4
Emilia Romagna	Early ALS	53 ± 3	2(M), 2(F)	2(S), 2(B)	43 ± 4	16 ± 3	4
	Early Controls	55 ± 3	2(M), 2(F)	n/a	n/a	n/a	4
	Late ALS	82 ± 5	3(M), 1(F)	4(B)	21 ± 11	16 ± 5	4
	Late Controls	83 ± 6	3(M), 1(F)	n/a	n/a	n/a	4
Study population	Early ALS	50 ± 5	8(M), 8(F)	11(S), 5(B)	28 ± 10	15 ± 4	16
	Early Controls	50 ± 6	8(M), 8(F)	n/a	n/a	n/a	16
	Late ALS	80 ± 4	8(M), 8(F)	7(S), 6(B), 3(–)	14 ± 6	15 ± 5	16
	Late Controls	79 ± 5	8(M), 8(F)	n/a	n/a	n/a	16

*^a^Age: age at peripheral blood mononuclear cells (PBMC) collection; ^b^Form: spinal (S) or bulbar (B) form of amyotrophic lateral sclerosis (ALS); ^c^Score: ALS Functional Rating Scale Revised at PBMC collection; ^d^Duration: disease duration (months) from the onset of symptoms to PBMC collection; n/a: not applicable; –: data not available. Values are expressed as mean ± SD.*

**Table 2:** Demographic and clinical characteristics of the enrolled population in the validation study.

Residency	Diagnosis	Age^a^	Sex	Form^b^	Score^c^	Duration^d^	*N*
Lombardia	Early ALS	47 ± 6	9(M), 6(F)	13(S), 2(B)	36 ± 6	21 ± 19	15
	Early Controls	47 ± 7	9(M), 7(F)	n/a	n/a	n/a	16
	Late ALS	78 ± 8	8(M), 9(F)	10(S), 7(B)	27 ± 10	21 ± 14	17
	Late Controls	78 ± 5	7(M), 9(F)	n/a	n/a	n/a	16
Piemonte	Early ALS	48 ± 4	6(M), 1(F)	7(S)	37 ± 10	21 ± 22	7
	Early Controls	48 ± 8	6 (M), 1(F)	n/a	n/a	n/a	7
	Late ALS	80 ± 4	7(M), 9(F)	10(S), 6(B)	32 ± 9	17 ± 17	16
	Late Controls	80 ± 6	7(M), 9(F)	n/a	n/a	n/a	16
Emilia Romagna	Early ALS	46 ± 7	6(M), 10(F)	11(S), 5(B)	33 ± 11	38 ± 17	16
	Early Controls	45 ± 9	8(M), 4(F)	n/a	n/a	n/a	12
	Late ALS	82 ± 6	6(M), 8(F)	7(S), 7(B)	27 ± 12	17 ± 10	14
	Late Controls	80 ± 6	8(M), 8(F)	n/a	n/a	n/a	16
Study population	Early ALS	47 ± 6	21(M), 17(F)	31(S), 7(B)	29 ± 20	29 ± 20	38
	Early Controls	46 ± 8	23(M), 12(F)	n/a	n/a	n/a	35
	Late ALS	80 ± 5	31(M), 26(F)	27(S), 20(B)	28 ± 10	19 ± 14	47
	Late Controls	79 ± 5	22(M), 26(F)	n/a	n/a	n/a	48

*^a^Age: age at PBMC collection; ^b^Form: spinal (S) or bulbar (B) form of ALS; ^c^Score: ALS Functional Rating Scale Revised at PBMC collection; ^d^Duration: disease duration (months) from the onset of symptoms to PBMC collection; n/a: not applicable. Values are expressed as mean ± SD.*

### Mice

Procedures involving animals and their care were approved by the Institutional Animal Care and Use Committee and were conducted in conformity with the following laws, regulations, and policies governing the care and use of laboratory animals: Italian Governing Law (D.lgs 26/2014; Authorisation n.19/2008-A issued March 6, 2008 by Ministry of Health); Mario Negri Institutional Regulations and Policies providing internal authorization for persons conducting animal experiments (Quality Management System Certificate – UNI EN ISO 9001:2008 – Reg. No. 6121); the NIH Guide for the Care and Use of Laboratory Animals (2011 edition) and EU directives and guidelines (EEC Council Directive 2010/63/UE). The Statement of Compliance (Assurance) with the Public Health Service (PHS) Policy on Human Care and Use of Laboratory Animals has been recently reviewed (9/9/2014) and will expire on September 30, 2019 (Animal Welfare Assurance #A5023-01). Animals were bred and maintained at the IRCCS-Istituto di Ricerche Farmacologiche Mario Negri, Milan under standard conditions, temperature 21 ± 1°C, relative humidity 55 ± 10%, 12h light schedule, food and water ad libitum. Transgenic SOD1^G93A^ mice expressing about 20 copies of mutant human SOD1 with a Gly93Ala substitution (B6SJL-TgSOD1G93A-1Gur) were originally obtained from Jackson Laboratories and maintained on a C57BL/6JOlaHsd (C57) genetic background at Harlan Italy S.R.L., Bresso, Milan, Italy. By crossbreeding C57 SOD1^G93A^ mice with 129S2/SvHsd (129Sv) mice for >15 generations, SOD1^G93A^ mice on a 129Sv homogenous background were generated (Marino et al., 2015). For biochemical analyses, female mice were deeply anesthetized with ketamine hydrochloride (IMALGENE, 150 mg/kg, Alcyon Italia S.p.A.) and medetomidine hydrochloride (DOMITOR, 2 mg/kg, Alcyon Italia S.p.A.) by intraperitoneal injection and euthanized by decapitation at symptom onset: for 129Sv SOD1^G93A^ mice at 14 weeks of age, and for C57 SOD1^G93A^ mice at 17 weeks of age. The corresponding age-matched nontransgenic mice were used as controls.

### PBMC Isolation and Protein Extraction

Peripheral blood mononuclear cells were isolated from peripheral venous blood of human subjects essentially as previously described (Nardo et al., 2011). Briefly, samples of blood were collected in EDTA pre-coated tubes (BD Vacutainer K2EDTA) and PBMC were isolated from EDTA blood by Ficoll-Hypaque (Ficoll-PlaqueTM Plus, GE Healthcare) density gradient centrifugation at 800 × *g* for 30 min at 18–20°C. Mononuclear cells were collected from the interface and washed three times with RPMI 1640 medium (EuroClone). Platelets were eliminated by an additional wash and centrifugation at 200 × *g* for 10 min. PBMC were stored as pellets at –80°C. PBMC from mice were isolated from blood sampled by intracardiac puncture and collected in EDTA pre-coated vials (BD Vacutainer K2EDTA). PBMC were isolated from EDTA-blood by Lympholite (Lympholyte-Mammal, Cedarlane) density gradient centrifugation at 1000 × *g* for 25 min at 18–20°C. Mononuclear cells were harvested from the interface and washed once at 800 × *g* for 10 min with RPMI 1640 (EuroClone) supplemented with 2.5 mM EDTA, and stored as pellets at –80°C. Before analysis human and mouse PBMC were lysed in 20 mM Tris-HCl pH 7.5, 0.1% NP40, and 0.1% SDS supplemented with Protease Inhibitors (Sigma), boiled for 5 min and centrifuged at 16.000 × *g* for 10 min at 4°C. Supernatants were analyzed by Western and dot blot analyses. Proteins were quantified by the BCA protein assay (Pierce).

### Protein Extraction from the Spinal Cord of the Mice

The spinal cord was flushed from the vertebral column and sectioned into cervical, thoracic and lumbar segments. The samples were immediately frozen on dry-ice and stored at –80°C until analysis. Lumbar spinal cords were homogenized by sonication in 1% boiling SDS. Protein homogenates were further boiled for 10 min and centrifuged at 13.500 × *g* for 5 min at 4°C. Supernatants were analyzed by Western and dot blot analyses. Proteins were quantified by the BCA protein assay (Pierce).

### Two-Dimensional Difference in Gel Electrophoresis (2D DIGE)

Peripheral blood mononuclear cells proteins were prepared for 2D DIGE analysis as follows: four pools of 20 μg from 16 early ALS, 16 early controls, 16 late ALS, and 16 late controls were methanol-precipitated overnight. Proteins were then dissolved in 30 mM Tris-HCl pH 8.5, 7 M urea, 2 M thiourea, CHAPS 4% (w/v) and Cydye-labeled according to the manufacturer’s instructions (GE Healthcare) with minor modifications. Briefly, 20 μg of each pool was labeled with 200 pmol of Cy3 or Cy5 dye for 30 min in ice in the dark. To exclude preferential labeling of the dyes, each sample was also reverse labeled. As an internal standard, aliquots of each pool were mixed and labeled with Cy2 dye. Labeled samples were then resuspended in Destreak SolutionTM (GE Healthcare) with IPG buffer pH 3-11 NL 1% v/v (GE Healthcare) and loaded into 11 cm-IPG strips pI range 3-11NL (GE Healthcare). Isoelectrofocusing was done on an IPGphor apparatus (GE Healthcare) with the following protocol: 300 Vhrs at 30 V, 50 Vhrs at 200 V, 1500 Vhrs at 2000 V, 2800 Vhrs of a linear gradient up to 3500 V, 4800 Vhrs at 3500 V, 8500 Vhr of a linear gradient up to 8000 V, and 30000 Vhr at 8000 V. SDS-PAGE was done using Precast Tris-HCl 10% polyacrylamide SDS gel (Biorad). Eight 2D gels were run with the four experimental groups. Each gel contained two experimental groups, one Cy3-labeled, the other Cy5-labeled plus the Cy2-labeled internal standard. Gel images were captured by the laser scanner Molecular Imager FX (Bio-Rad). Image analysis was done with Progenesis Same Spot software (Nonlinear Dynamics). For each spot the normalized volume was standardized against internal standard spot normal volume. The values for each spot in each group were expressed as the mean of the Cy3- and Cy5-labeled analyses. Values for early and late ALS were further normalized to the means values of early and late controls, respectively. Differential spots were considered only those that had a fold change (normalized late ALS/ normalized early ALS) ≤0.8 or ≥1.3.

### Protein Identification

Differential protein spots were located and excised from 2D gels with the EXQuest^TM^ spot cutter (Bio-Rad). Spots were processed and gel-digested with modified trypsin from bovine pancreas (Roche) and identified by mass spectrometry (MS), as previously described (Nardo et al., 2011). Peptide mass fingerprinting and tandem MS/MS were done on a 4800 MALDI TOF/TOF mass spectrometer (Applied Biosystems). The mass spectra were internally calibrated with trypsin autolysis fragments. The five most abundant precursor ions, out of the exclusion mass list (ions from human keratin and trypsin), were selected for MS/MS analysis. The combined MS and MS/MS data were submitted by GPS Explorer v.3.6 software (Applied Biosystems) to the MASCOT database search engine (Version 2.1, Matrix Science) and searched with the following parameters: Uniprot_Swissprot 2012x database over all Homo *sapiens* protein sequences deposited, no fixed modifications, as possible modifications carboamidomethylation of cysteine and oxidation of methionine, 1 missed trypsin cleavage, a mass tolerance of ±0.1 Da for the peptide masses and ±0.3 Da for the MS/MS fragment ion masses. A protein was regarded as identified if the MASCOT protein score, based on the combined MS and MS/MS data, was above the 5% significance threshold for the database (Pappin et al., 1993).

### Gene Ontology (GO) Analysis

Identified proteins were classified on the basis of gene ontology (GO) annotations provided by PANTHER^1^ (Mi et al., 2013). PANTHER overrepresentation test was performed with the list of the identified differential proteins against the whole *Homo sapiens* reference list and against a list of about 800 proteins extracted from published works of 2D-based proteomics of lymphocytes/monocytes (Verhoeckx et al., 2004; Rosengren et al., 2005; Xie et al., 2005; Azkargorta et al., 2006; Han et al., 2006; Huang et al., 2006; Ramirez-Boo et al., 2006; Salonen et al., 2006; Zada et al., 2006; Henrich et al., 2007; Lai et al., 2007; Rakkola et al., 2007; Skopeliti et al., 2007; Vergara et al., 2008). PANTHER GO-Slim Biological Process and GO-Slim Molecular Function were used as annotation data sets (version 11.1, released 2016-07-15).

### Dot Blot Analysis

Dot blot was used in the validation study after verification that the antibody detected specific bands in WB (Supplementary Figure S1). Proteins (3 μg) were directly loaded onto nitrocellulose Trans-Blot transfer 0.2–0.45 μm (Bio-Rad) membranes, depositing each sample on the membrane by vacuum filtration, as described previously (Nardo et al., 2011). An internal standard which is a pool of all samples in the analysis (healthy controls and patients) was deposited in triplicates. Dot blot membranes were blocked with 3% (w/v) BSA (Sigma) and 0.1% (v/v) Tween 20 in Tris-buffered saline, pH 7.5, incubated with primary antibodies, then with peroxidase-conjugated secondary antibodies (Santa Cruz Biotechnology Inc.). Blots were developed with Luminata^TM^ Forte Western Chemiluminescent HRP Substrate (Millipore) on the ChemiDoc XRS system (Bio-Rad). Densitometry was done with Progenesis PG240 v2006 software (Nonlinear Dynamics). The immunoreactivity of the different proteins was normalized to Ponceau Red staining (Fluka) and to the internal standard of each membrane.

### Statistical Analysis

Immunoreactivity values for each protein analyzed in early and late ALS were normalized to the means values of early and late controls, respectively. The statistical analysis of the protein expression in the two groups was done by Student’s *t*-test using PRISM software (GraphPad, San Diego, CA, USA, version 6.01).

## Results

### Identification of Candidate Phenotypic Biomarkers of ALS in PBMC by 2D-DIGE-Based Proteomics

**FIGURE 1:**
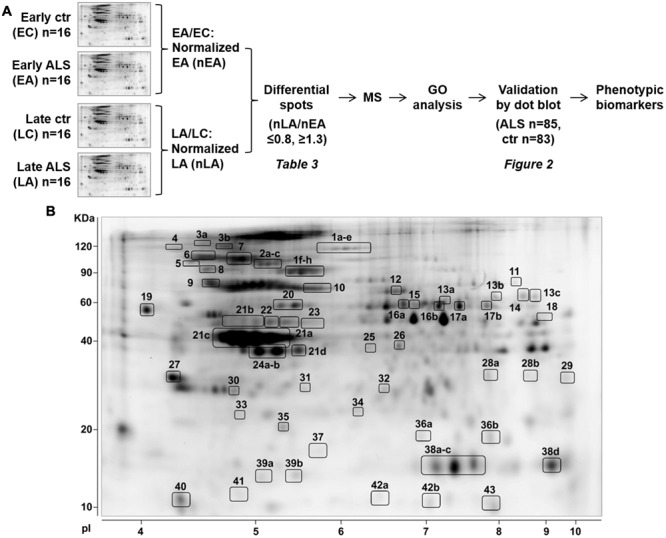
**Identification of phenotypic biomarkers in PBMC of early and late ALS patients. (A)** Scheme of the strategy to identify phenotypic protein biomarkers in PBMC of patients early onset ALS (EA) and late-onset ALS (LA) and matched controls, early (EC), and late (LC). 2D-DIGE analysis was done with 16 pooled samples for each experimental group and spot volume values for EA and LA were normalized to EC and LC, respectively (nEA, nLA). Spots were considered differential if fold change (nLA/nEA) was ≤0.8 or ≥1.3 and identified by mass spectrometry (MS, Supplementary Table S1). Differential proteins were classified on the basis of GO annotations and a selected number of proteins associated with “protein folding and response to stress” biological function was validated by dot blot immunoassays. **(B)** Representative Sypro-Ruby stained 2D gel of the PBMC proteome. The numbered spots correspond to the differential proteins listed in **Table 3** and Supplementary Table S1.

**Figure 1A** schematically shows the strategy we used to identify and validate phenotypic protein biomarkers of ALS in PBMC. In the discovery phase, PBMC of ALS patients with ≤55 years of age (early ALS, EA) or ≥75 years of age (late ALS) at diagnosis and matched early and late controls were analyzed by 2D DIGE. The analysis was done with 16 pooled samples for each of the four experimental groups (**Table 1**). Spot volume values for early and late ALS were normalized to the mean values of early and late controls, respectively. Spots were considered differential if fold change (normalized late ALS/normalized early ALS) was ≤0.8 or ≥1.3. A total of 68 differential spots were detected (**Figure 1B**). From these spots 43 unique proteins were identified by MS as candidate protein biomarkers (**Table 3**). Some of the proteins were identified in multiple spots at different pI and Mw, which are fragments or post-translationally modified isoforms of the same protein (Supplementary Table S1). We grouped the differential proteins on the basis of their most recognized function and on GO annotations (**Table 3**). The overrepresentation test performed with the list of the differential proteins against the *Homo sapiens* reference list indicated isomerase activity (GO:0016853), associated with PPIA and ERp57, among the most significantly enriched (*p* = 0.03; fold = 7.8) molecular functions. Protein folding (GO:0006457) and response to stress (GO:0006950) were among the most significantly enriched (*p* < 0.01; fold>4) biological processes identified. The overrepresentation test performed also against a reference list of lymphocyte/monocyte proteins, identified in 2D-based proteomic studies, indicated response to stress as one of the most significantly enriched (*p* = 0.03; fold = 2.5) biological processes. This suggested that proteins associated with these molecular functions and biological processes (**Table 3**), such as PPIA, HSP90, GRP78, ERp57, and DJ-1, were attracting candidate phenotypic biomarkers to further validate in a larger and well-characterized cohort of patients with early and late disease onset.

**Table 3:** Differential spots.

Spot	Uniprot	Protein name	Fold change
**Cytoskeleton-associated proteins**			
1a-h	P18206	Vinculin	1.6
2a-c	P06396	Gelsolin	2.1
7	P12814	Alpha-actinin-1	1.6
12	O75083	WD repeat-containing protein 1	1.3
21a-d	P63261	Actin, cytoplasmic 2	1.8
22	P60709	Actin, cytoplasmic 1	1.4
24a-b	P52907	F-actin-capping protein subunit alpha-1	0.7
**Protein folding and response to stress**			
5	P14625	Endoplasmin	1.4
8	P07900	**Heat shock protein HSP 90-alpha (HSP90)**	1.3
9	P11021	**78 kDa glucose-regulated protein (GRP78)**	1.3
19	P27797	Calreticulin	1.3
20	P30101	**Protein disulfide-isomerase A3 (ERp57)**	1.3
32	P30041	Peroxiredoxin-6	0.7
33	P09211	Glutathione S-transferase P	0.8
34	Q99497	**Protein deglycase DJ-1 (DJ-1)**	0.7
36a-b	P62937	**Peptidyl-prolyl *cis-trans* isomerase A (PPIA)**	0.8
29	P23396	40S ribosomal protein S3	0.6
43	P02775	Platalet basic protein (CXCL7)	1.3
**Metabolic processes**			
11	P29401	Transketolase	0.8
13a-c	P14618	Pyruvate kinase isozymes M1/M2	1.4
14	P14619	Pyruvate kinase isozymes M1/M3	1.3
15a-b	P11413	Glucose-6-phosphate 1-dehydrogenase	1.3
16	P00367	Glutamate dehydrogenase 1, mitochondrial	1.4
25	P11172	Uridine 5′-monophosphate synthase	0.7
31	O95336	6-Phosphogluconolactonase	1.3
**Inflammatory response**			
39a-b	P06702	Protein S100 A9	0.7
42a-b	P05109	Protein S100 A8	0.6
26	P04083	Annexin A1	0.6
**Signal transduction**			
27	P63104	14-3-3 protein zeta/delta	1.3
28a	Q15404	Ras suppressor protein 1	0.7
28b	Q15404	Ras suppressor protein 1	1.3
30	P52566	Rho GDP-dissociation inhibitor 2	0.7
**Others**			
3a	P07996	Thrombospondin-1	0.7
3b	P07996	Thrombospondin-1	1.7
4	P35442	Thrombospondin-2	1.5
6	P08514	Integrin alpha-IIb	1.5
18	P52272	Heterogeneous nuclear ribonucleoprotein M	1.3
35	P37802	Transgelin-2	1.3
37	P61088	Ubiquitin-conjugating enzyme E2 N	0.5
40	Q9H299	SH3 domain-binding glutamic acid-rich-like protein 3	1.3
41	P58546	Myotrophin	0.7
**Blood contaminant**			
10	P02768	Serum albumin	1.3
17a-b	P02671	Fibrinogen beta chain	1.4
23	P02679	Fibrinogen gamma chain	1.9
38a-d	P68871	Hemoglobin subunit beta	0.6

*Uniprot: entry from the UniProt Knowledgebase database; Fold change: spot volume fold change (nLA/nEA), in case of multiple spots of the same protein with a similar trend, mean fold change is reported. Mass spectrometry (MS) data for each spot are reported in Supplementary Table S1. In bold the proteins analyzed in the validation study.*

### Validation of Candidate Phenotypic Biomarkers

**FIGURE 2:**
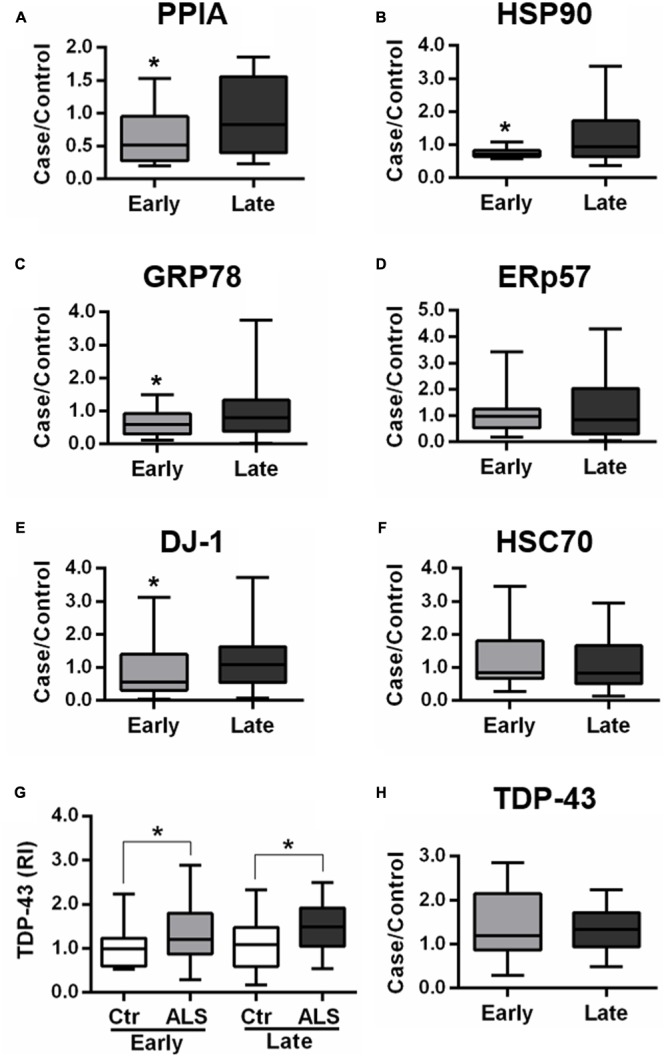
**Validation of candidate phenotypic biomarkers in PBMC of early and late ALS patients. (A–H)** PPIA, HSP90, GRP78, ERp57, DJ-1, HSC70, and TDP-43 were analyzed by dot blot immunoassays in PBMC samples from an independent set of ALS patients (*n* = 85), *n* = 38 EA and *n* = 47 LA, and matched controls (*n* = 83), *n* = 35 EC and *n* = 48 LC. Immunoreactivity was normalized to protein loading, as assessed by Ponceau Red staining, and then to the mean values of matched controls **(A–F,H)**. We found that PPIA, HSP90, GRP78, and DJ-1 were significantly lower in early ALS than in late ALS **(A–C,E)** and that TDP-43 was higher in both early and late ALS compared to matched controls **(G)**. ^∗^*p* < 0.05, by Student’s *t*-test.

In the validation study, we analyzed total protein levels of candidate phenotypic biomarkers, PPIA, HSP90, GRP78, ERp57, and DJ-1, by dot blot analysis in PBMC samples from an independent set of ALS patients (*n* = 85), *n* = 38 early ALS and *n* = 47 late ALS, and matched controls (*n* = 83), *n* = 35 early controls and *n* = 48 late controls (**Table 2**). Immunoreactivity values for each protein in early and late ALS patients were normalized to the mean values of early and late controls, respectively, similarly to the proteomic analysis. We found that PPIA, HSP90, GRP78, and DJ-1 were significantly lower in early ALS than in late ALS, and therefore can be considered phenotypic biomarkers (**Figures 2A–C,E**), while ERp57 did not change (**Figure 2D**). Moreover, PPIA, HSP90, GRP78, and DJ-1 were similarly regulated, with a decreased level in early ALS compared to early controls and an opposite tendency in late ALS (Supplementary Figures S2A–C,E), suggesting that early ALS patients have some defects in the response to stress pathways. In the same validation analysis, we measured another chaperone protein, HSC70, and TDP-43, both not differential in the 2D-DIGE analysis, but up-regulated in PBMC of a cohort of ALS patients with a mean age of onset of 62 ± 10 years, previously reported in a work by our laboratory (Nardo et al., 2011). We found that HSC70 could not distinguish early from late ALS (**Figure 2F**), but it was up-regulated in late ALS compared to controls (Supplementary Figure S2F). We confirmed that total TDP-43 protein levels in PBMC underscores disease (**Figure 2G**), in fact it was higher in both early and late ALS compared to controls, but could not discriminate early ALS from late ALS (**Figure 2H**). It has to be noted that in our assay the total TDP-43 protein level comprise the 43-kDa full-length and fragmented forms (Supplementary Figure S1A).

The experimental set up of the analysis has strengths and limitations. The major strengths are the population base and the enrolment of patients with newly diagnosed ALS. Our patients are a fairly representative sample of an incident ALS population. The major limitation is the small sample size. The analysis may thus be underpowered for some differences to achieve statistical significance. Then, we did not adjust our data for multiple comparisons. Thus, we cannot exclude that some of the associations are chance findings. However, due to the exploratory nature of the analysis, we deliberately decided not to use the Bonferroni correction for multiple testing.

### Analysis of Phenotypic Biomarkers in Two SOD1^G93A^ Mouse Models with Different Age of Onset

**FIGURE 3:**
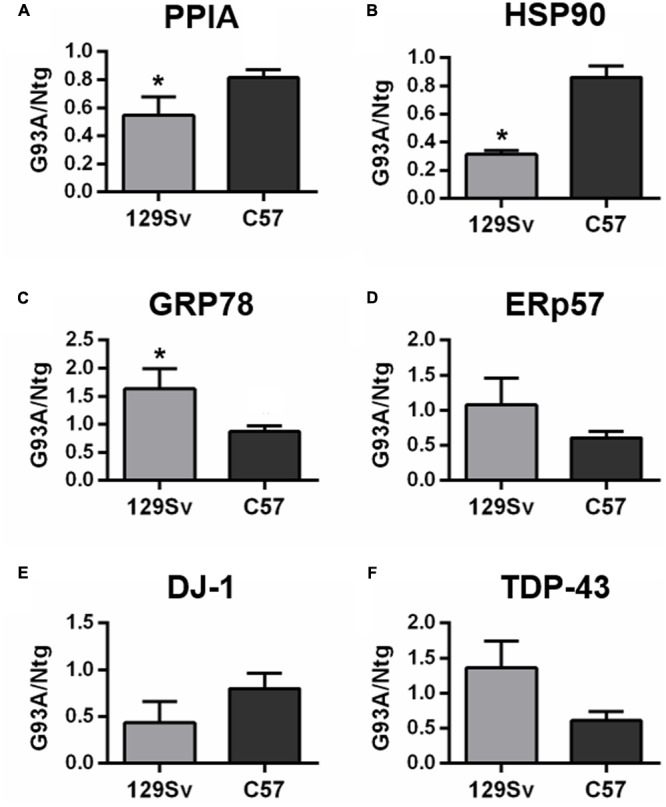
**Analysis of phenotypic biomarkers in PBMC of SOD1^G93A^ mouse models with early (129Sv) and late (C57) disease onset. (A–F)** PPIA, HSP90, GRP78, ERp57, DJ-1, and TDP-43 were analyzed by dot blot immunoassays in PBMC samples (*n* = 5 per group) from 129Sv SOD1^G93A^ and C57 SOD1^G93A^ (G93A) mice at disease onset, respectively, at 14 and 17 weeks of age, and matched non-transgenic controls (Ntg). Immunoreactivity was normalized to protein loading, as assessed by Ponceau Red staining, and then to the mean values of matched controls. We found that PPIA, HSP90 were significantly lower in early ALS than in late ALS **(A,B)** similarly to human samples. ^∗^*p* < 0.05, by Student’s *t*-test.

We also analyzed total protein levels of the candidate phenotypic biomarkers, PPIA, HSP90, GRP78, ERp57, and DJ-1 in PBMC of two SOD1^G93A^ mouse models, 129Sv SOD1^G93A^ and C57 SOD1^G93A^ mice and corresponding non-transgenic controls by dot blot analysis. These mice despite having equal SOD1^G93A^ expression have a different disease phenotype, because of the different genetic background (Marino et al., 2015). In particular, 129Sv and C57 SOD1^G93A^ mice have symptom onset at 14 and 17 weeks of age, respectively, thus representing our mouse models of early (129Sv) and late (C57) disease onset. Similarly, to the human samples, immunoreactivity values for each protein in early and late mouse models were normalized to the mean values of the corresponding non-transgenic controls. We found that PPIA and HSP90 were significantly lower in early SOD1^G93A^ mice than in late SOD1^G93A^ mice, as much as in the human samples (**Figures 3A,B**), and there was a significant lower level of the two proteins in the early SOD1^G93A^ mice compared to their non-transgenic controls (Supplementary Figures S3A,B). DJ-1 had a similar behavior but it did not reach statistical significance (**Figure 3E** and Supplementary Figure S3E). GRP78 was instead significantly higher in early SOD1^G93A^ mice than in late SOD1^G93A^ mice (**Figure 3C**), in contrast with the human samples, with an increased level in the early SOD1^G93A^ mice compared to controls (Supplementary Figure S3C). ERp57 was not significantly different in late and early SOD1^G93A^ mice, as much as in the human samples (**Figure 3D**), but it was significantly lower in late SOD1^G93A^ mice compared to controls (Supplementary Figure S3D). Finally, TDP-43 was not significantly different in early and late SOD1^G93A^ mice, as in the human samples (**Figure 3F**), but was not different compared to corresponding nontransgenic controls, in contrast with the human samples (Supplementary Figure S3F).

**FIGURE 4:**
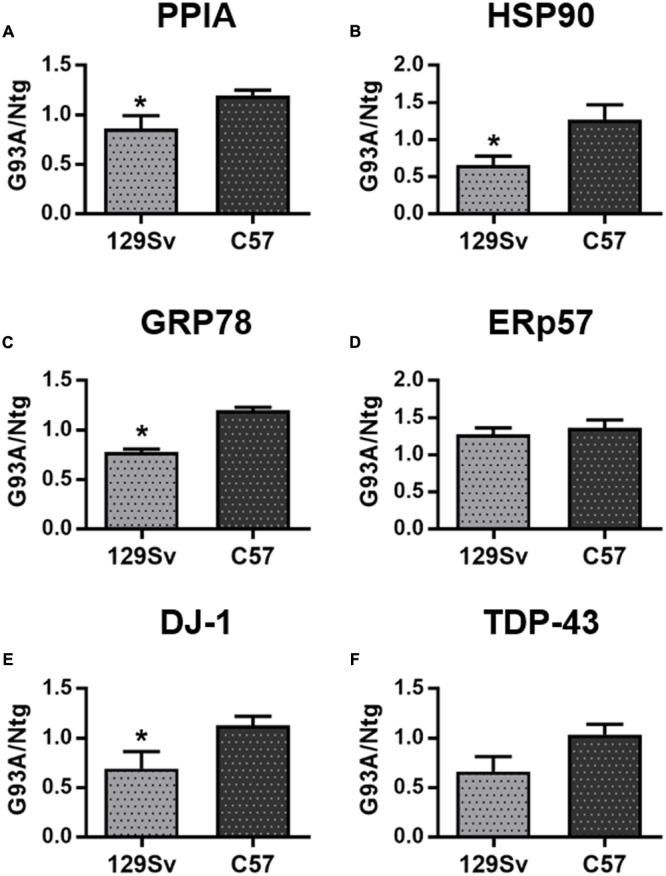
**Analysis of phenotypic biomarkers in lumbar spinal cord of SOD1^G93A^ mouse models with early (129Sv) and late (C57) disease onset. (A–F)** PPIA, HSP90, GRP78, ERp57, DJ-1, and TDP-43 were analyzed by dot blot immunoassays in lumbar spinal cord samples (*n* = 5 per group) from 129Sv SOD1^G93A^ and C57 SOD1^G93A^ (G93A) mice at disease onset, respectively, at 14 and 17 weeks of age, and matched non-transgenic controls (Ntg). Immunoreactivity was normalized to protein loading, as assessed by Ponceau Red staining, and then to the mean values of matched controls. We found that PPIA, HSP90, GRP78, and DJ-1 were significantly lower in early ALS than in late ALS **(A–C,E)** similarly to PBMC from patients. ^∗^*p* < 0.05, by Student’s *t*-test.

Total protein levels of PPIA, HSP90, GRP78, DJ-1, and TDP-43 were also measured in lumbar spinal cord homogenates from the early and late SOD1^G93A^ mice at disease onset and relative age-matched nontransgenic controls by dot blot analysis (**Figure 4** and Supplementary Figure S4). PPIA, HSP90, GRP78 and DJ-1 were significantly lower in early SOD1^G93A^ mice than in late SOD1^G93A^ mice (**Figures 4A–C,E**) and were similarly regulated to PBMC isolated from patients (Supplementary Figures S4A–E), indicating that also early SOD1^G93A^ mice have some defects in the response to stress pathways, as already reported (Nardo et al., 2013, 2016; Marino et al., 2015). ERp57 and TDP-43 were not different in the two conditions as in the human samples (**Figures 4D,F**). These data confirmed a previous work where no differences in total soluble TDP-43 were found between the SOD1^G93A^ mice and respective Ntg littermates and between the two mouse strains (Marino et al., 2015). In conclusion, we observed a similar regulation of these proteins, in PBMC of patients and PBMC and spinal cord of mice (**Table 4**).

**Table 4:** Summary of the biomarker analysis in early and late ALS.

SAMPLES	PPIA	HSP90	GRP78	ERp57	DJ-1	TDP-43
Human PBMC	↓	↓	↓	↔	↓	↔
Mouse PBMC	↓	↓	↑	↔	↔	↔
Mouse spinal cord	↓	↓	↓	↔	↓	↔

*↓, significantly lower in early onset disease; ↔, not significantly different; ↑, significantly higher in early onset disease.*

## Discussion

The molecular determinants of ALS heterogeneity are still poorly understood. An anticipated or a delayed onset of the disease may be caused by various combinations of intrinsic and exogenous factors converging into common pathogenic pathways that underlie different susceptibility to ALS and eventually lead to a different prognosis. Here we report the identification of protein biomarkers in PBMC that can discriminate ALS patients with an early disease onset from those with a late disease onset. The identified phenotypic biomarkers have in common a high expression in CNS where they have an important role in protein folding, as foldases and/or classical molecular chaperones, and are upregulated by a variety of stressors. They participate at different levels and in different ways in the maintenance of proteostasis, whose deficit has shown to generate protein misfolding and aggregation and facilitate the development of several neurodegenerative diseases, including ALS (Ruegsegger and Saxena, 2016). These phenotypic biomarkers are: PPIA, HSP90, GRP78, and DJ-1.

Polyclonal anti-peptidyl-prolyl *cis-trans* isomerase A is ubiquitously expressed, with the highest expression in neurons and motor neurons (Lauranzano et al., 2015). It is a peptidylprolyl cis/trans isomerase and, as a foldase, it accelerates the rate-limiting steps along the folding pathway, but it also acts as a classical molecular chaperone (Fischer et al., 1989; Freskgard et al., 1992). We originally identified it as a candidate protein biomarker in PBMC of ALS patients with classical disease onset, where it is upregulated also in association with disease progression (Nardo et al., 2011). We next found that mutant SOD1 and TDP-43 are substrates of PPIA (Lauranzano et al., 2015). In fact, in absence of PPIA increased levels of mutant SOD1 and TDP-43 were recovered in the aggregates isolated from the spinal cord of SOD1^G93A^ mice that had also an anticipation of the onset and an acceleration of the disease progression.

HSP90 is one of the most abundant and conserved cytosolic heat shock proteins. It is responsible for the correct folding of a number of newly synthesized proteins and for the refolding of misfolded proteins associated with neurodegenerative diseases, including TDP-43 (Basso et al., 2009; Daturpalli et al., 2013; Carlomagno et al., 2014; Schirmer et al., 2016). Accordingly, we found it entrapped in the aggregates isolated from the spinal cord of SOD1^G93A^ mice already at a presymptomatic stage of the disease and from spinal cord tissues of sporadic ALS patients (Basso et al., 2009).

GRP78 is a major ER chaperone and a key regulator of the unfolded protein response. Disturbance of ER homeostasis is a common feature of ALS and defects in ER chaperones caused motor dysfunction in experimental models of ALS (Atkin et al., 2008; Saxena et al., 2009; Woehlbier et al., 2016). Loss of one copy of SIL1, a GRP78 cofactor, anticipated disease onset and reduced life span in the SOD1^G93A^ mouse model (Filézac de L’Etang et al., 2015), however, the precise role of GRP78 in ALS remains to be determined. In Alzheimer’s disease, studies *in vitro* indicated that it may be part of the defense mechanisms of the cell by inhibiting the generation of amyloid-β peptides (Yang et al., 1998; Hoshino et al., 2007).

DJ-1 is a multifunctional stress response protein with a strong and homogenous expression in all CNS regions (Bader et al., 2005). DJ-1 mutations cause early onset autosomal recessive Parkinson’s disease (Bonifati et al., 2003). It is a chaperone for alpha synuclein and was shown to be protective against oxidative stress by upregulating HSP70 (Shendelman et al., 2004; Zhou et al., 2006; Batelli et al., 2008). In the SOD1^G93A^ mouse model increased oxidized/inactive DJ-1 forms were found in brain tissues and knocking out DJ-1 has led to an accelerated disease course and shortened survival time (Lev et al., 2009, 2015).

Interestingly, we found that PPIA, HSP90, GRP78, and DJ-1 are present at lower levels in PBMC from patients with an early onset disease, and have a similar behavior in PBMC and spinal cord of an early onset SOD1^G93A^ mouse model, in which defects in protein quality control have been previously observed (Marino et al., 2015). These proteins, both in patient PBMC and mouse samples, are less upregulated in early ALS than in late ALS, and for PPIA this is evident already at a presymptomatic stage of the disease in the soluble fraction of the mouse spinal cord (Marino et al., 2015). The bases of these differences in early and late-onset disease are solely genetic and yet unknown in the case of the SOD1^G93A^ mouse models, but exogenous factors cannot be excluded in the patients. For example, it has been reported that exposure to certain toxins lead to GRP78 downregulation (Yang et al., 2000; Namba et al., 2010).

## Conclusion

These data suggest that a failure in the response to stress and a reduced ability to upregulate protective proteins may increase susceptibility to ALS. This is in agreement with the fact that vulnerable motor neurons have a high threshold for induction of the protective heat shock response and a higher sensitivity to ER stress (Batulan et al., 2003; Saxena et al., 2009). A protein signature comprising a panel of proteins involved in proteostasis may underline a subset of patients more susceptible to the disease and may help to stratify patients for more targeted ALS clinical trials. These observations further strengthen the notion that proteostasis maintenance by the ubiquitin–proteasome system, autophagy and ER, is a central issue in ALS, and therapeutic approaches aiming at boosting the protein quality control system might be a promising therapeutic strategy. Arimoclomol, a small molecule that acts as a co-inducer of the heat shock response by prolonging HSF1 activation and upregulating a number of proteins such as HSP60, HSP70, HSP90, and GRP94, has shown to be effective in several experimental models of motor neuron degeneration including the SOD1^G93A^ mouse (Kalmar et al., 2014). A Phase II clinical trial in ALS patients has shown that the drug is safe and well tolerated (Cudkowicz et al., 2008) and is now under investigation in a PhaseII/III clinical trial in SOD1 positive familial ALS patients (ClinicalTrials.gov identifier: NCT00706147). Colchicine, a FDA-approved drug that was identified by a high-throughput screening as enhancer of the expression of HSPB8, a key player of the protein quality control system, has demonstrated to facilitate the removal of TDP-43 aggregates by autophagy (Crippa et al., 2016).

Finally, this work confirmed that PBMC are valuable clinical samples since they reflect traits of the disease observed in the central nervous system and that total TDP-43 protein level in PBMC can discriminate ALS patients from healthy controls, as observed in a previous work using the same assay (Nardo et al., 2011). We also further confirmed that the experimental setting in which both 129Sv and C57 SOD1^G93A^ mouse strains are examined is useful to study disease susceptibility and test novel therapeutic approaches aimed at addressing disease heterogeneity.

## Ethics Statement

This study was carried out in accordance with the GCP recommendations with written informed consent from all subjects. All subjects gave written informed consent in accordance with the Declaration of Helsinki. The protocol was approved by the Ethics Committees of the participating institutions.

Procedures involving animals and their care were conducted in conformity with the following laws, regulations, and policies governing the care and use of laboratory animals: Italian Governing Law (D.lgs 26/2014; Authorisation n.19/2008-A issued March 6, 2008 by Ministry of Health); Mario Negri Institutional Regulations and Policies providing internal authorization for persons conducting animal experiments (Quality Management System Certificate – UNI EN ISO 9001:2008 – Reg. No. 6121); the NIH Guide for the Care and Use of Laboratory Animals (2011 edition) and EU directives and guidelines (EEC Council Directive 2010/63/UE). The Statement of Compliance (Assurance) with the Public Health Service (PHS) Policy on Human Care and Use of Laboratory Animals has been recently reviewed (9/9/2014) and will expire on September 30, 2019 (Animal Welfare Assurance #A5023-01).

## Author Contributions

Study concept and design: EB, CB, VB. Acquisition, analysis, or interpretation of data: all authors. Drafting the manuscript: VB. Critical revision of the manuscript: EB, CB.

## Conflict of Interest Statement

The authors declare that the research was conducted in the absence of any commercial or financial relationships that could be construed as a potential conflict of interest.

## Footnotes

1 http://www.pantherdb.org/
