# Supplementary material for: Decreased Levels of Foldase and Chaperone Proteins Are Associated with an Early-Onset Amyotrophic Lateral Sclerosis
Source: Front Mol Neurosci. 2017 Apr 6;10:99. doi: 10.3389/fnmol.2017.00099 (PMC5382314; doi:10.3389/fnmol.2017.00099)
Supplement: Supplementary file 1 [file Data_Sheet_1.pdf]

Supplementary Material

Decreased levels of foldase and chaperone proteins are associated with an early-onset amyotrophic lateral sclerosis

Melania Filareti, Silvia Luotti, Laura Pasetto, Mauro Pignataro, Katia Paolella, Paolo Messina, Elisabetta Pupillo, Massimiliano Filosto, Christian Lunetta, Jessica Mandrioli, Giuseppe Fuda, Andrea Calvo, Adriano Chiò, Massimo Corbo, Caterina Bendotti, Ettore Beghi, Valentina Bonetto*

*** Correspondence: Valentina Bonetto:** valentina.bonetto@marionegri.it

Supplementary Table 1. Differential spots identified by mass spectrometry.

Spot	Fold	Uniprot	Protein name	Mw_{cal}	pI_{cal}	Mw_{obs}	pI_{obs}	Coverage	Peptides	Score
1a	1.5	P18206	Vinculin	124	5.5	120	6.1	48	60	279
1b	1.6	P18206	Vinculin	124	5.5	120	5.7	49	69	295
1c	1.5	P18206	Vinculin	124	5.5	120	5.7	61	96	429
1d	1.5	P18206	Vinculin	124	5.5	120	5.8	49	63	313
1e	1.5	P18206	Vinculin	124	5.5	120	5.8	37	40	109
1f	1.9	P18206	Vinculin	124	5.5	92.4	5.2	52	49	183
1g	1.8	P18206	Vinculin	124	5.5	92.4	5.2	52	49	197
1h	1.7	P18206	Vinculin	124	5.5	92.4	5.5	49	62	197
2a	1.8	P06396	Gelsolin	280	5.7	85.6	5.7	35	30	127
2b	2.0	P06396	Gelsolin	280	5.9	85.6	5.9	48	43	188
2c	2.1	P06396	Gelsolin	280	5.9	85.6	5.9	59	55	279
3a	0.7	P07996	Thrombospondin-1	129	4.7	130	4.7	47	48	143
3b	1.7	P07996	Thrombospondin-1	129	4.7	130	4.9	22	28	130
4	1.5	P35442	Thrombospondin-2	129	4.6	130	4.6	39	39	91
5	1.4	P14625	Endoplasmin	92.4	4.8	90	4.9	63	67	326
6	1.5	P08514	Integrin alpha-IIb	113	5.2	110	4.7	36	46	271
7	1.6	P12814	Alpha-actinin-1	103	5.3	100	5.2	64	76	208
8	1.3	P07900	Heat shock protein HSP 90-alpha	84.6	4.9	90	4.9	58	51	103
9	1.3	P11021	78 kDa glucose-regulated protein	72.3	5.1	75	4.7	56	49	469

10	1.3	P02768	Serum albumin	69.3	5.9	70	5.9	54	49	148
11	0.8	P29401	Transketolase	67.8	7.6	70	8.5	44	24	127
12	1.3	O75083	WD repeat-containing protein 1	66.1	6.2	70	6.5	58	33	113
13a	1.5	P14618	Pyruvate kinase isozymes M1/M2	57.9	8.0	60	7.2	75	52	177
13b	1.3	P14618	Pyruvate kinase isozymes M1/M2	57.9	8.0	60	8.0	73	48	157
13c	1.4	P14618	Pyruvate kinase isozymes M1/M2	57.9	8.0	60	9.0	73	45	152
14	1.3	P14619	Pyruvate kinase isozymes M1/M3	57.9	8.0	60	8.5	83	61	186
15	1.4	P00367	Glutamate dehydrogenase 1, mitochondrial	61.3	7.7	60	7.0	39	26	90
16a	1.3	P11413	Glucose-6-phosphate 1-dehydrogenase	59.2	6.4	60	6.5	55	27	110
16b	1.3	P11413	Glucose-6-phosphate 1-dehydrogenase	59.2	6.4	60	7.0	64	41	112
17a	1.3	P02671	Fibrinogen beta chain	55.9	8.5	60	7.5	60	41	108
17b	1.4	P02671	Fibrinogen beta chain	55.9	8.5	60	7.7	57	32	80
18	1.3	P52272	Heterogeneous nuclear ribonucleoprotein M	77.4	8.8	60	9.0	36	44	63
19	1.3	P27797	Calreticulin	48.1	4.3	60	4.2	57	22	141
20	1.3	P30101	Protein disulfide-isomerase A3	56.7	6.0	60	5.5	49	34	180
21a	1.9	P63261	Actin, cytoplasmic 2	41.7	5.3	45	5.0	54	30	267
21b	1.5	P63261	Actin, cytoplasmic 2	41.7	5.3	45	5.0	52	21	100
21c	1.6	P63261	Actin, cytoplasmic 2	41.7	5.3	42	5.0	71	34	317
21d	1.4	P63261	Actin, cytoplasmic 2	41.7	5.3	41	5.0	43	19	174
22	1.4	P60709	Actin, cytoplasmic 1	41.7	5.3	42	5.0	40	16	92
23	1.9	P02679	Fibrinogen gamma chain	51.5	5.4	45	5.6	67	37	130
24a	0.7	P52907	F-actin-capping protein subunit alpha-1	32.9	5.5	35	5.3	86	26	124
24b	0.7	P52907	F-actin-capping protein subunit alpha-1	32.9	5.5	35	5.3	77	24	125

25	0.7	P11172	Uridine 5'-monophosphate synthase	52.1	6.8	42	6.3	40	24	61
26	0.6	P04083	Annexin A1	38.7	6.6	40	6.7	51	19	155
27	1.3	P63104	14-3-3 protein zeta/delta	27.7	4.7	30	4.4	81	37	262
28a	0.7	Q15404	Ras suppressor protein 1	31.5	8.6	30	8.0	38	13	59
28b	1.3	Q15404	Ras suppressor protein 1	31.5	8.6	30	8.5	38	12	61
29	0.6	P23396	40S ribosomal protein S3	26.7	9.7	30	10	65	19	122
30	0.7	P52566	Rho GDP-dissociation inhibitor 2	22.9	5.1	25	4.9	79	20	82
31	1.3	O95336	6-Phosphogluconolactonase	27.5	5.7	25	5.6	63	17	76
32	0.7	P30041	Peroxiredoxin-6	27.5	5.7	25	6.5	63	17	76
33	0.8	P09211	Glutathione S-transferase P	23.3	5.4	22	4.9	65	15	151
34	0.7	Q99497	Protein DJ-1	19.8	6.3	22	6.2	56	18	67
35	1.3	P37802	Transgelin-2	22.3	8.4	20	5.3	88	24	137
36a	0.7	P62937	Peptidyl-prolyl cis-trans isomerase A	18.0	7.7	18	7.0	66	14	57
36b	0.8	P62937	Peptidyl-prolyl cis-trans isomerase A	18.0	7.7	18	8.0	47	15	106
37	0.5	P61088	Ubiquitin-conjugating enzyme E2 N	17.1	6.1	17	5.8	69	14	78
38a	0.6	P68871	Hemoglobin subunit beta	15.9	6.7	16	7.0	95	20	302
38b	0.6	P68871	Hemoglobin subunit beta	15.9	6.7	16	7.3	31	7	188
38c	0.5	P68871	Hemoglobin subunit beta	15.9	6.7	16	7.8	85	18	234
38d	0.5	P68871	Hemoglobin subunit beta	15.9	6.7	16	9.0	85	21	326
39a	0.7	P06702	Protein S100 A9	13.2	5.7	13	5.2	86	12	120
39b	0.6	P06702	Protein S100 A9	13.2	5.7	13	5.4	90	16	130
40	1.3	Q9H299	SH3 domain-binding glutamic acid-rich-like protein 3	10.4	4.8	12	4.4	40	7	79

41	0.7	P58546	Myotrophin	12.8	5.3	12	4.8	53	7	65
42a	0.5	P05109	Protein S100 A8	10.8	6.5	11	6.4	76	15	182
42b	0.6	P05109	Protein S100 A8	10.8	6.5	11	7.0	63	15	192
43	1.3	P02775	Platalet basic protein (CXCL7)	13.8	9.1	12	8.0	55	11	106

Fold: normalized late/early ALS fold change; Uniprot: entry from the UniProt Knowledgebase database; Mw_{calc} and pI_{calc}, calculated Mw and pI; Mw_{obs} and pI_{obs}, observed Mw and pI; Coverage: % sequence coverage; Peptides: number of peptides identified; Score: MASCOT protein score derived from the combination of MS and MS/MS data. Proteins were identified based on combined MS and MS/MS on a 4800 MALDI TOF/TOF mass spectrometer (Applied Biosystems)

Supplementary Figures

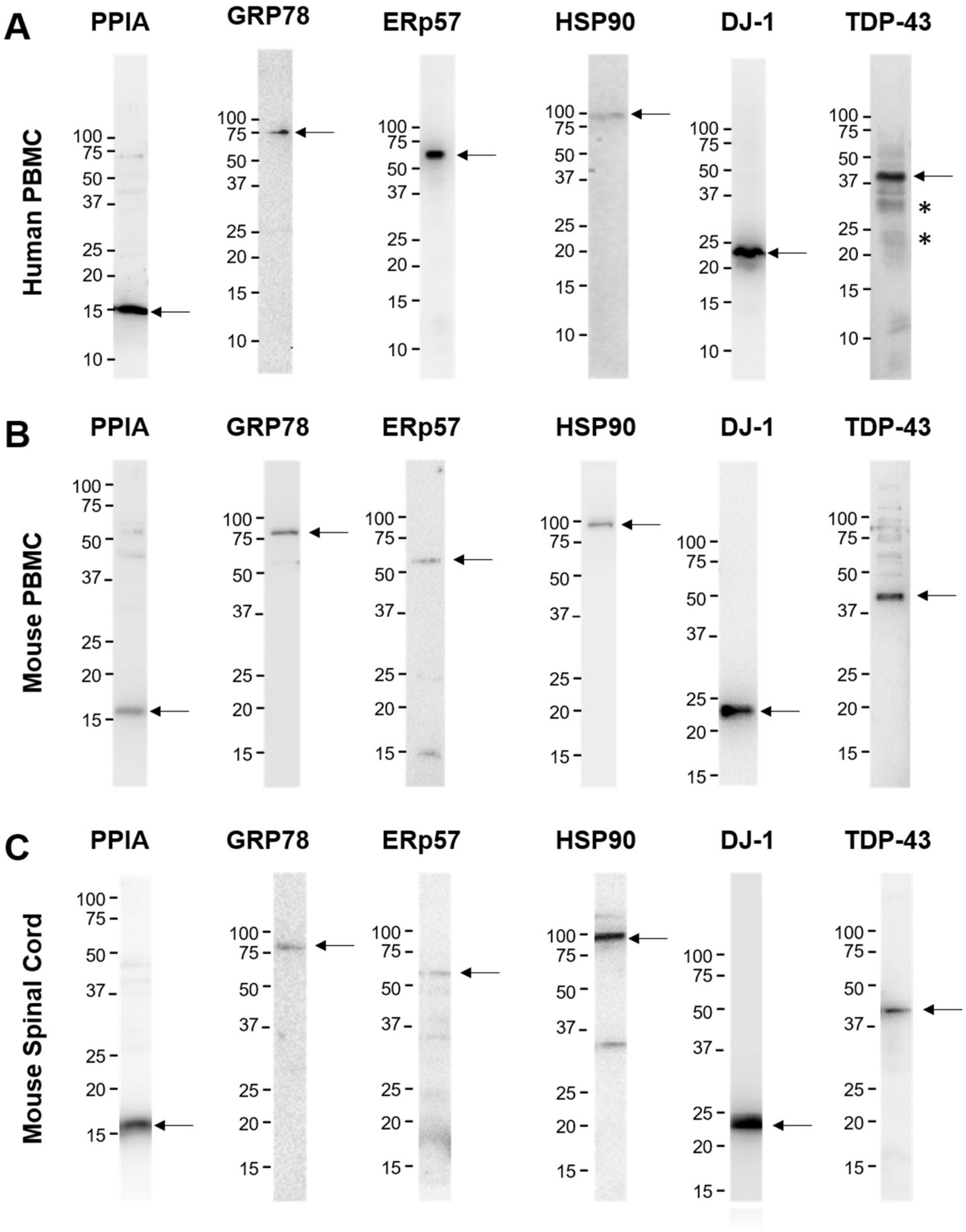

Supplementary Figure 1. The setting up of the dot blot immunoassays for human and mouse samples. Western blot analysis of human PBMC (A), mouse PBMC (B) and spinal cord (C) lysates were done using primary antibodies for PPIA, HSP90, GRP78, ERp57, DJ-1 and TDP-43 as reported in the Material and Method section. All Western blots showed a major single band at the expected molecular weight indicating that the antibodies were specific and could be used in the dot blot assay. For human PBMC the internal standard, which is a pool of all samples in the analysis (healthy controls and patients), was analyzed. Asterisks indicate fragmented TDP-43.

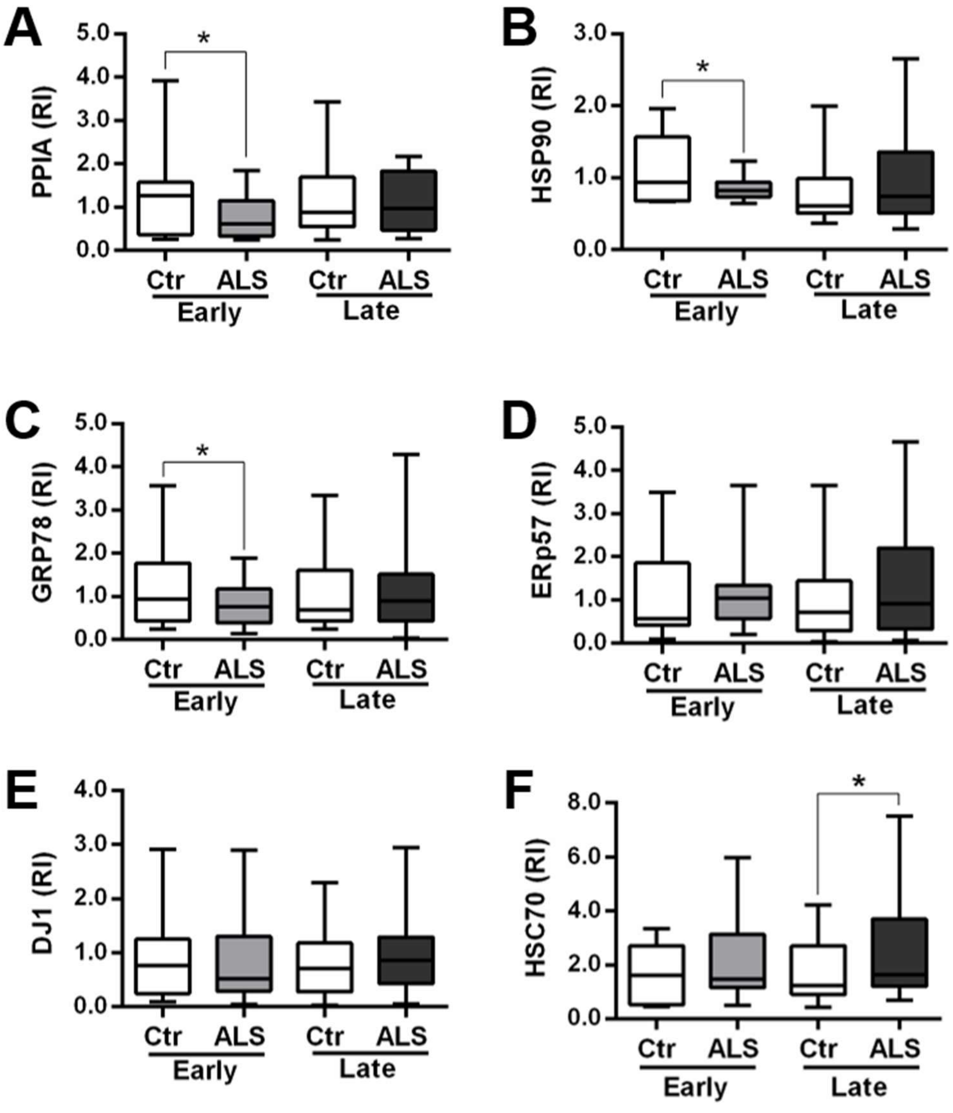

Supplementary Figure 2. Validation of candidate phenotypic biomarkers in PBMC of early and late ALS patients. (A-F) PPIA, HSP90, GRP78, ERp57, DJ-1, HSC70 were analyzed by dot blot immunoassays in PBMC samples from an independent set of ALS patients (n=85), n=38 EA and n=47 LA, and matched controls (n=83), n=35 EC and n=48 LC. Immunoreactivity was normalized to protein loading, as assessed by Ponceau Red staining (Relative immunoreactivity, RI). We found that PPIA, HSP90, GRP78 were significantly lower in early ALS than in matched controls (A-C) and that HSC70 was higher in late ALS compared to matched controls (F). *, $p < 0.05$, by Student's t test.

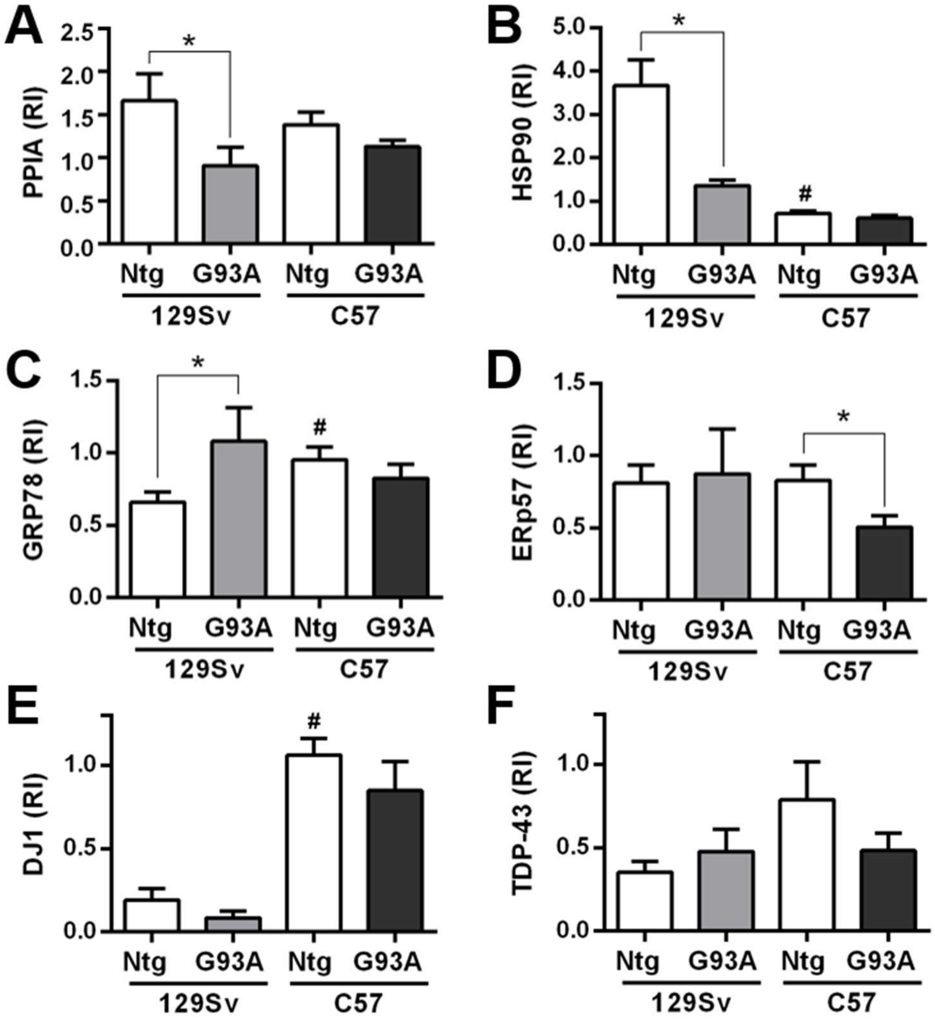

Supplementary Figure 3. Analysis of phenotypic biomarkers in PBMC from SOD1^{G93A} mouse models with early (129Sv) and late (C57) disease onset. (A-F) PPIA, HSP90, GRP78, ERp57, DJ-1, and TDP-43 were analyzed by dot blot immunoassays in PBMC samples (n= 5 per group) from 129Sv SOD1^{G93A} and C57 SOD1^{G93A} (G93A) mice at disease onset, respectively at 14 and 17 weeks of age, and matched nontransgenic controls (Ntg). Immunoreactivity was normalized to protein loading, as assessed by Ponceau Red staining (Relative immunoreactivity, RI). We found that PPIA, HSP90 were significantly lower in early ALS mice compared to nontransgenic controls (A-B), GRP78 was significantly higher in early ALS mice compared to nontransgenic controls (C), ERp57 was significantly lower in late ALS mice compared to nontransgenic controls (D). *, $p < 0.05$, by Student's t test; #, $p < 0.05$ versus nontransgenic 129Sv, by Student's t test.

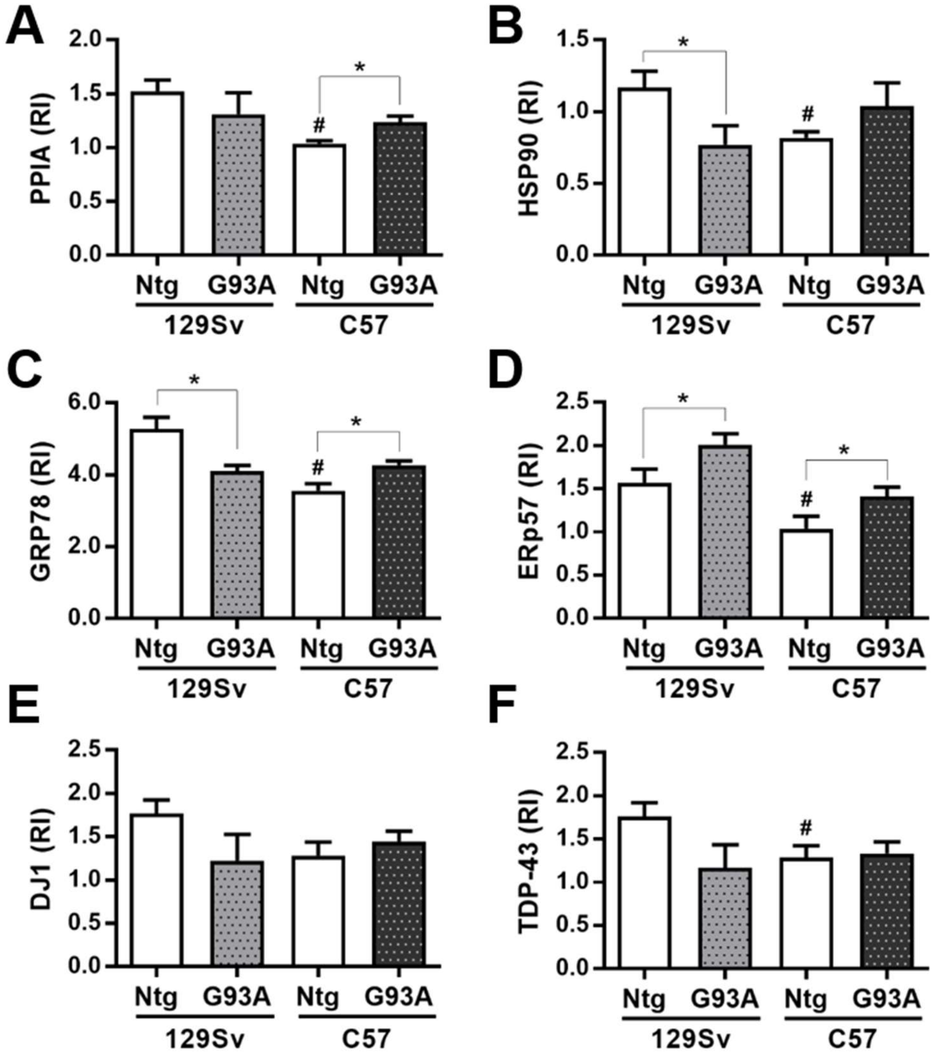

Supplementary Figure 4. Analysis of phenotypic biomarkers in lumbar spinal cord from SOD1^{G93A} mouse models with early (129Sv) and late (C57) disease onset. (A-F) PPIA, HSP90, GRP78, ERp57, DJ-1, and TDP-43 were analyzed by dot blot immunoassays in lumbar spinal cord samples (n= 5 per group) from 129Sv SOD1^{G93A} and C57 SOD1^{G93A} (G93A) mice at disease onset, respectively at 14 and 17 weeks of age, and matched nontransgenic controls (Ntg). Immunoreactivity was normalized to protein loading, as assessed by Ponceau Red staining (Relative immunoreactivity, RI). We found that PPIA, GRP78 and ERp57 were significantly higher in late ALS mice compared to nontransgenic controls (A,C,D), HSP90 and GRP78 was significantly lower in early ALS mice compared to nontransgenic controls (B-C), ERp57 was significantly higher in early ALS mice compared to nontransgenic controls (D). *, $p < 0.05$, by Student's t test; #, $p < 0.05$ versus nontransgenic 129Sv, by Student's t test.
